# Apoptosis-mediated anti-proliferative activity of *Calligonum comosum* against human breast cancer cells, and molecular docking of its major polyphenolics to Caspase-3

**DOI:** 10.3389/fcell.2022.972111

**Published:** 2022-10-10

**Authors:** Ashok Kumar, Mohammad A. Alfhili, Ahmed Bari, Hanane Ennaji, Maqusood Ahamed, Mohammed Bourhia, Mohamed Chebaibi, Laila Benbacer, Hazem K. Ghneim, Manal Abudawood, Khalid M. Alghamdi, John P. Giesy, Yazeed A. Al-Sheikh, Mourad A. M. Aboul-Soud

**Affiliations:** ^1^ Vitiligo Research Chair, Department of Dermatology, College of Medicine, King Saud University, Riyadh, Saudi Arabia; ^2^ Chair of Medical and Molecular Genetics Research, Department of Clinical Laboratory Sciences, College of Applied Medical Sciences, King Saud University, Riyadh, Saudi Arabia; ^3^ Department of Pharmaceutical Chemistry, College of Pharmacy, King Saud University, Riyadh, Saudi Arabia; ^4^ Laboratory of Chemistry, Biochemistry Nutrition, and Environment, Faculty of Medicine and Pharmacy, University Hassan II, Casablanca, Morocco; ^5^ King Abdullah Institute for Nanotechnology, King Saud University, Riyadh, Saudi Arabia; ^6^ Biomedical and Translational Research Laboratory, Faculty of Medicine and Pharmacy of the Fez, University of Sidi Mohamed Ben Abdellah, Fez, Morocco; ^7^ Research Unit and Medical Biology, National Center for Nuclear Energy, Science and Technology, CNESTEN, Rabat, Morocco; ^8^ Department of Dermatology, College of Medicine, King Saud University, Riyadh, Saudi Arabia; ^9^ Toxicology Centre, University of Saskatchewan, Saskatoon, SK, Canada; ^10^ Department of Veterinary Biomedical Sciences, University of Saskatchewan, Saskatoon, SK, Canada; ^11^ Department of Integrative Biology, Michigan State University, East Lansing, MI, United States; ^12^ Department of Environmental Sciences, Baylor University, Waco, TX, United States

**Keywords:** Calligonum comosum, cytotoxicity, anticancer, gas chromatography mass spectrometry, apoptosis, natural products, fire bush, molecular docking

## Abstract

Due to poor diagnosis breast cancer in women has emerged as the most common cause of death disease in developing countries. Medicinal plants have been used for thousands of years and can be useful in healthcare, especially in developing countries. Ethanol extracts of leaves of fire bush or arta (*Calligonum comosum;* EECC), exhibited significant anticancer potencies against two breast cancer cell lines, MCF-7 and MDA 231. These *in vitro* effects of EECC indicated potential anticancer activities that were determined to be specific since minimal toxicity was recorded against MCF-12, a non-cancerous breast cell line used as a reference. EECC also induced cell cycle arrest in MCF-7 and MDA 231 as revealed by the increased proportions of sub-G1 cells. Fluorescence-activated cell sorter analysis (FACS), utilizing double staining by annexin V-FITC/propidium iodide, revealed that the observed cytotoxic effects were mediated *via* apoptosis and necrosis. FACS measurement of thegreater in fluorescence intensity, linked with oxidation of DCFH to DCF, revealed that apoptosis was attributable to production of free radicals. EECC-mediated apoptosis was further validated by observation of up-regulation in the “executioner” enzyme, caspase 3. The current findings reveal that EECC exhibits significant, selective cytotoxicity to breast cancer cells, that proceeds *via* the generation of ROS, which culminates in apoptosis. The anti-proliferative effects of EECC weres further verified by use of a structure-based, virtual screening between its major bioactive polyphenolic constituents and the apoptosis executioner marker enzyme, caspase-3. Based on their glide score values against the active site of caspase 3, some phyto-constituents present in EECC, such as DL-alpha-tocopherol and campesterol, exhibited distinctive, drug-like potential with no predicted toxicity to non-target cells. Taken together, the usefulness of natural phenolic and flavonoid compounds contained in *Calligonum comosum* were suggested to be potent anticancer agents.

## Introduction

Cancer, a devastating and aggressive disease, is a leading source of human suffering and fatality worldwide and its prevalence is increasing. Cancer is already one of the most commonly reported ailments, despite of development of treatments including chemotherapeutic drugs. One in four deaths in the United States is due to cancer. Breast cancer known as the record widespread potentially fatal disease in women, is the fifth most frequent cause of death in developed countries, but due to insignificant diagnosis in developing countries is the second leading cause of death (Ghoncheh et al., 2016). Inadequacy of surgery and the serious adverse side effects of standard anticancer therapeutic strategies make development of natural anticancer drugs an attractive alternative, especially in developing countries where, due to costs and the need for advanced facilities, other therapies might be less available (Mitra and Dash, 2018; Varghese et al., 2018). Plant-derived anticancer drugs have both preventative and therapeutic potentials (Iqbal et al., 2017). Because of the toxicity profile of existing therapies, as well as the significant danger of initiating uncontrollable inflammatory responses caused by deceased cancerous cells, a variety of natural phytochemicals are employed as alternative or complementary therapies (Cheng et al., 2018).

Plants of the genus *Calligonum* comprise around 80 species that are found throughout North Africa, Europe, West and Central Asia (Boulos, 2000). The fire bush or “arta” (*Calligonum comosum*) is a tiny shrub with small leaves, that is a member of the Polygonaceae family, which is called an acid family. *Calligonum comosum* is an Egyptian plant that grows in deserts and is found in tropical places. Moreover, it is used for tanning leather and as a smokeless source of fuel. Furthermore, it is utilized as a medicinal herb in conventional folklore medicine, particularly in rural regions, to cure stomach disorders and toothache (Ghazanfar, 1994). *Calligonum comosum* has also been shown to be effective against skin disorders and as antibacterial, anti-inflammatory, anti-ulcer and anticancer agents (Liu et al., 2001; Badria et al., 2007; Riadh et al., 2011). Recently, the methanolic extract derived from fruit hairs of *Calligonum comosum*, collected from the Saudi desert, has been reported to exhibit strong antioxidant and anticancer properties against human hepatocarcinoma cells (Alzahrani, 2021). All parts of *Calligonum comosum* were found to contain turpines triple antrakinont and kumarinat substances, flavonoids and alkaloids (Alzahrani, 2021).

Investigation of effects of natural and synthetic antitumor compounds, using established tumor cell lines, have achieved outstanding results. Because of the lesser toxicity compared to currently used therapeutics, various natural products from plant sources are being used as an alternative to multiple cancers (Schwartsmann, 2001; Aboul-Soud et al., 2020). Recent innovative, technological breakthroughs in computer sciences as well as accessibility to structures of proteins and small molecules have aided in development of new methodologies. *In silico* models, such as molecular docking, are increasingly popular in both industrial and academic contexts (Safwat et al., 2021; Roy et al., 2022). In the current study, cytotoxic and anti-proliferative activities of ethanolic extracts of leaves of *Calligonum comosum* (EECC) on two breast cancer cell lines, MCF-7 and MDA-MB-231, were investigated. The mode-of-action of the observed anticancer activity was investigated by use of FACS analysis of annexin V-FITC/propidium iodide dual staining. In addition, molecular docking of the major bioactive constituents in EECC against the binding site of the apoptosis “executioner enzyme,” caspase 3, was conducted.

## Materials and methods

### Chemicals

All chemicals and reagents were of molecular biology grade and purchased from Sigma-Aldrich (St Louis, MO, United States). Analytical grade ethanol was purchased from Merck Chemical Inc. (Darmstadt, Germany). DMEM (Dulbecco’s modified eagle medium), antibiotic-antimycotic (penicillin and streptomycin), _L_-glutamine and FBS (Fetal Bovine Serum) were obtained from Gibco Inc. (NY, United States). Tissue culture flasks (25 and 75 cm^2^) with vent neck, filtered cap, sterile 15 ml- and 50-ml centrifuge polystyrene tubes, individually-wrapped sterile polystyrene pasture serological pipettes were obtained from Corning® USA.

### Source of *Calligonum comosum*

Plant material used during this study was leaves of *Colligonum comosum*. Specimens collected, during the flowering stage, in February 2020 from sandy habitat near Agadir City, southern Atlantic coast (29.602956°N - 9.006312°W) before being identified by Prof. Fennane Mohammed (Scientific Institute, Mohammed V University, Rabat, Morocco) with help of the main floristic guides; i.e., Practical Flora of Morocco (Fennane et al., 1999, 2007 and 2014). Subsequently, the specimen was assigned the voucher code RAB112037.

### Extraction of *Calligonum comosum* leaves

Fresh and healthy leaves of *Calligonum comosum* were cleaned under running water, then air-dried at room temperature (RT) in the laboratory, out of direct sun light for 15 days. Afterward, dried leaves were turned into fine powder by use of an electric grinder. Powder was kept at 4°C in a stainless steel bottle with 1 g silica pouch to prevent moisture. Powdered air-dried *Calligonum comosum* leaves (10 g) were sonicated for 30 min at RT and then extracted two times using 250 ml by 95% ethanol each. Total 500 ml supernatant pooled together and centrifuge it at 6000 × *g* for 15 min. Evaporate it at 40°C under reduced pressure to get solvent free ethanol extract. Dried extract was stored until further use at 4°C. Stock solution was prepared by adding DMSO in dried extract to perform biological assays.

### Cell lines and culturing conditions

MDA-MB-231 and MCF-7 breast cancer cell lines utilized in the current study were initially purchased from the American Type Culture Collection, USA. Breast cancer lines were maintained in full medium of DMEM medium supplemented with high glucose, 10% FBS, 1% antibiotic-antimycotic at 37°C incubator provided with 5% CO_2_ and 90% humidity in 25 and 75 cm^2^ cell culture flasks. For the anti-proliferative assay, cancer cells were trypsin-treated by use of Trypsin 0.05%/0.53 mM EDTA solution, and reaction was stopped by adding full medium and seeded into 96-well plates (Gibco, United States) as 2 × 10^3^ cell/well in 200 µl of DMEM for 24 h prior to exposure to increasing concentrations of EECC for additional 48 h as explained previously (Aboul-Soud et al., 2020).

### Anti-proliferative activity assessed by MTT assay

Cytotoxicity of EECC was evaluated by use of MTT, 3-[4,5-dimethylthiazol-2-yl]-2,5-diphenyltetrazolium bromide, assay, which was conducted in 96-well plates. MTT based viabilities were measured at 24, 48 and 72 h of incubation with several concentrations, including 15.6, 31.25, 62.5, 125, 250, 250 or 1000 µg EECC/ml, the treatment medium was discarded, and 100 µl per well of MTT solution (0.5 mg/ml, dissolved in PBS) was added to adherent cells and then kept at 37°C for further 3 h at 37°C as mentioned above. MTT solution was discarded from each well after incubation and 100 µl isopropanol was added in order to solubilize the purple crystals of formazan by shaking for an additional 2 h at room temperature. Afterward, absorbance at 549 nm, was read by use of a microplate reader (ELX 800, BioTek Instruments, Winooski, VT, United States). Exposures were done as triplicate and anti-proliferation expressed as percent. Concentrations causing 50% inhibition (IC_50_) of proliferation of breast cancerous cells (MCF-7 and MDA-MB-231) were calculated as described previously (Zafar et al., 2017; Aboul-Soud et al., 2020, 2022).

### Apoptosis/necrosis detection by flow cytometry

Apoptosis status of control and treated MCF-7 and MDA 231 breast cancer cells exposed to EECC were examined by use of the Annexin-V Apoptosis Detection Kit I (Thermo Fisher Scientific, MA, United States) by flow cytometry (Aboul-Soud et al., 2020). MCF-7 and MDA 231 breast cancer cells were treated with 75, 150, or 300 µg EECC/mL, and then incubated for 48 h as described above. Control without any treatment and treated breast cancer cells were collected by washing with PBS, and then stained with 1% Annexin-V-FITC in addition 20 μg/ml PI prepared in binding buffer and in dark at RT for 20 min. Following incubation, fluorescence of PI and Annexin-V-FITC was detected using FACS (Calibur, Betcon Dickinson, Franklin Lakes, NJ, United States) flow cytometer at 488/520 and 535/670 nm, respectively.

### Cell cycle analysis

Control and experimental cells were stained with 50 μg/ml PI in presence of 0.1 mg/ml RNase A for 30 min at room temperature in total darkness. Cells were subsequently analyzed for DNA content based on PI binding by flow cytometry (Chen et al., 2018).

### Detection of reactive oxygen species

Reactive oxidative species (ROS) was determined in breast cancer cells exposed to EECC, by use of 2′,7′-dichlorodihydrofuorescein diacetate (H_2_DCFDA; Solarbio Life Science, Beijing, China) was applied to measure intracellular ROS (Ashour et al., 2016). Briefly, control without any treatment and treated breast cancer cells were collected by washing with PBS and labeled by 10 µM H_2_DCFDA and incubated at 37°C for 30 min away from light. Following repeated washing, the resultant fluorescent compound, 2′,7′-dichlorofluorescein, was read at 488 nm (blue laser) and at 520 nm.

### Caspase-3 enzyme activity assay

Caspase-3 enzyme activity was examined in cell lysate obtained from control without any treatment and treated breast cancer cells exposed to EECC. MCF-7 breast cancer cells at a density of 5 × 10^4^ were plated in T25 culture flasks in medium containing 37.7, 75.5, 150 or 300 µg EECC/ml. A reaction mixture to perform caspase-3 enzyme activity contained 30 µl of cell lysate of treated breast cancer cells, caspase-3 substrate of (20 μl, Ac-DEVDAFC), HEPES (150 μl, 50 mM), 1 mM EDTA and 1 mM DTT, was incubated at pH 7.2. The reaction mixture was measured at with an excitation wavelength of 430 nm and emission wavelength of/535 nm, by use of a microplate reader (Synergy HT, Bio-Tek, Winooski, VT, United States) for 15 min at 5 min intervals. Standard 7-amido-4- trifluoromethylcoumarin (AFC) was prepared with concentration ranges of 5–15 μM, and fluorescence was read followed by the calculation of caspase-3 enzyme activity in picomoles AFC released/min/mg protein (Akhtar et al., 2020).

### Gas chromatography-mass spectrometry (GC–MS)

GC-MS analysis employed a gas chromatograph (Agilent GC 7890A) coupled to a triple axis detector 5975C with a single quadruple mass spectrometer. The chromatographic column specification carriers gas flow rate, injector temperature, the source and Quad temperatures of MS and oven temperature gradient have been described previously (Chebbac et al., 2022). The range of scanning was adjusted at 40–600 ion masses at electron energy of 70 eV and solvent delay time of 3 min. Phytochemicals were identified by comparing the obtained spectra with those provided by the National Institute of Standard and Technology library (NIST 2008). Single sample analysis time was approximately 55 min. Adams Library (Adams, 2007) was also employed to compare the obtained mass spectra of EECC phytochemicals were with those of similar library compounds.

### Molecular docking of EECC compounds in caspase-3 active site

For the molecular docking study, ligprep, prepwizard, grid generation and docking calculations were executed by using of Schrodinger Maestro software with the Glide standard precision (SP) module. The following parameters: Glide score, energy, emodel and ligand efficiency were predicted.

1. Ligand Preparation

All compounds (ligands) are downloaded from PUBCHEM in .SDF format. Ligands are prepared for docking calculations by use of the LigPrep tool present in the Maestro 11.5 version of the Schrödinger Software employing the OPLS3 force field. As a maximum, a total of 32 stereoisomers were selected for the ligand following the ionization states at pH 7.0.

2. Protein preparation

The caspase-3 (PDB:3GJQ) 3D crystal structure was downloaded in PDB format from PDB (protein data bank; El-Sheref et al., 2020). Next, refinement and preparation of the structure was conducted by use of the Protein Preparation Wizard in Schrödinger-Maestro v11.5. Bond orders and charges were assigned, hydrogens were attached to heavy atoms, selenomethionine were converted to methionine in the proteins and finally all remaining waters were removed. Force field OPLS3 was employed for the minimization by setting maximum heavy atom RMSD (root-mean-square-deviation) to the value of 0.30 Å.

3. Receptor Grid Generation

The creation module was operated by mouse clicking on any atom present in the ligand so that a default grid box is generated. The dimensions of grid box were adjusted to 20 × 20 × 20, in terms of volumetric spacing, whereas the coordinates were fixed at x: 28.642, y: 34.169 and z: 12.047. In the grid box generated, ligand was coupled to produce protein by use of Extra Precision (XP). Subsequently, XP GScore was used for the evaluation of results.

4. Glide Standard Precision (SP) ligand docking

Ligand docking of SP flexible was conducted by use of the Glide interface of Schrödinger-Maestro v 11.5; penalties were included for amide bonds (non-cis/trans) (Friesner et al., 2006; Balamurugan et al., 2012). Selection of Van der Waals scaling factor and partial charge cutoff for ligand atoms was set to 0.80 and 0.15, respectively. Energy-minimized orientations were used to conduct final scoring and Glide score was displayed. For each ligand, the best-docked orientation, having the minimum Glide score numerical value was determined.

### Statistical analysis

Data are presented as means ± SEM. Experiments were conducted three times each in duplicates. Flow cytometry data were analyzed by use of FlowJo^™^ Software v10.7.2 (Becton, Dickinson and Company, Ashland, OR, United States), and appropriate significance tests were used to analyze the data (Student *t*-test for two groups and one-way ANOVA for three or more) by use of Microsoft Excel (Microsoft Corp., Washington, United States) and GraphPad Prism v9.2.0 (GraphPad Software, Inc., CA, United States). A value of *p* <0.05 denoted cutoff for statistical significance.

## Results

### Effect of various concentrations of EECC on proliferation of breast cancer cell lines

**FIGURE 1:**
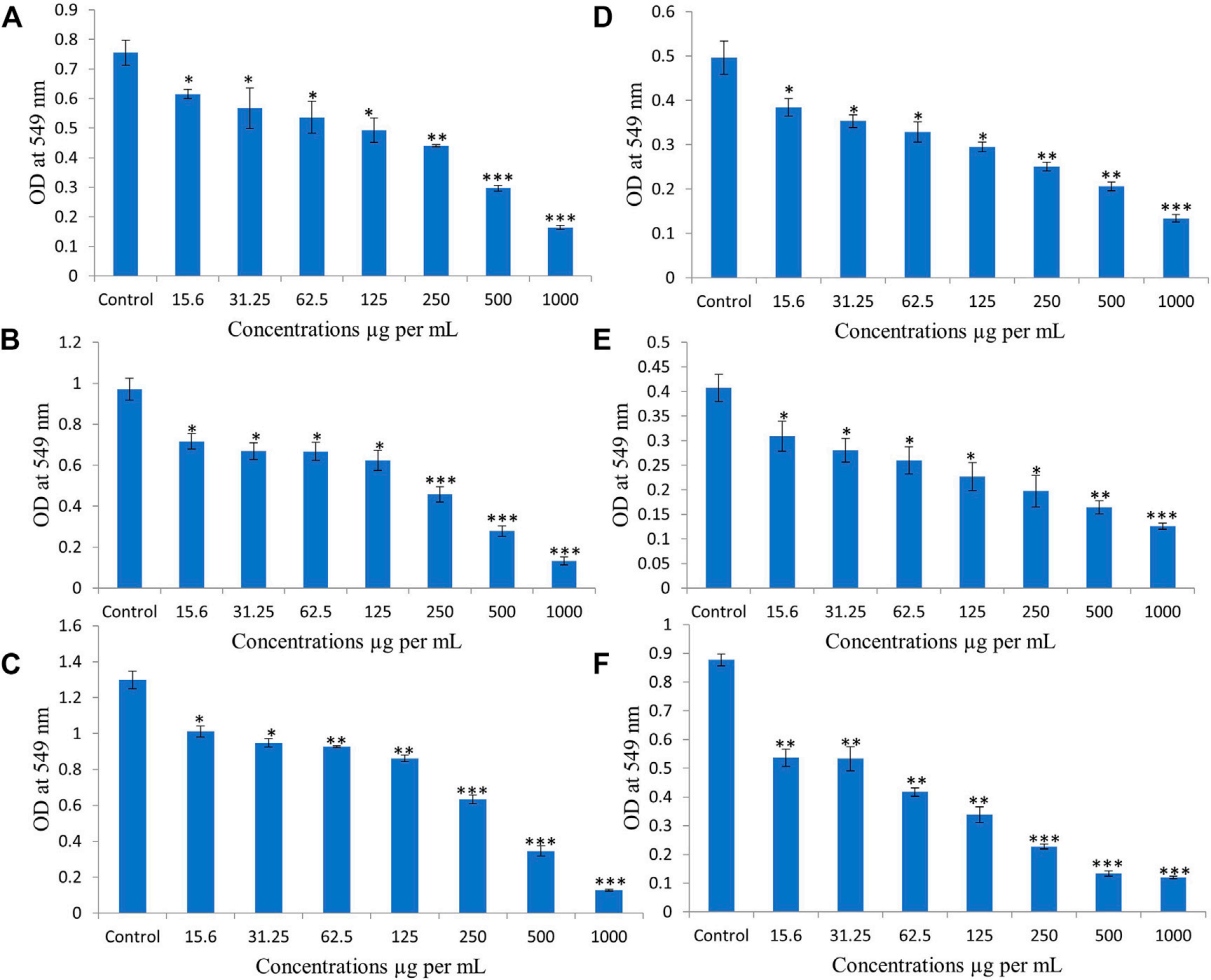
Ethanolic extract of *Colligonum comosum* (EECC) inhibits proliferation of two breast cancer cells, MCF-7 (**A**, for 24 h; **B**, for 48 h; **C**, for 72 h) and MDA 231 (**D**, 24 h; **E**, for 48 h; **F**, for 72 h). Cancer cells were treated with indicated concentrations of EECC with DMSO control for 24, 48 and 72 h followed by determination of proliferation by MTT assay, as detailed in the Materials and Methods part. *p < 0.05, **p < 0.01, ***p < 0.0001 vs. control. Data represent mean ± SD of eight technical well-replicates.

Two breast cancer cell lines, MCF-7 and MDA 231 were used to study anti-proliferative effects of EECC, by conducting a dose escalation study utilizing MTT-based viability assays. MCF-7 cells exposed to EECC showed concentration-dependent growth inhibition compared with control of 81, 75, 71, 65, 58, 39 and 21% when exposed for 24 h; 73, 68, 68, 64, 47, 28 and 13% when exposed for 48 h; 78, 73, 71, 66, 48, 26 and 9% for 72 h to 15.6, 31.25, 62.5, 125, 250, 500 or 1000 µg EECC/ml, respectively (Figures 1A–C). Anti-proliferative effects of EECC also exhibited concentration-dependent inhibition of growth of MDA 231 compared with control of 77, 71, 66, 59, 51, 41 and 27% when exposed for 24 h; 75, 68, 63, 55, 48, 40 and 30% when exposed for 48 h; 61, 60, 47, 38, 25% 15 and 13% when exposed to 72 h to 15.6, 31.25, 62.5, 125, 250, 500 or 1000 µg EECC/ml, respectively (Figures 1D–F). Concentrations resulting in 50% inhibition of growth of cancer cells (IC_50_), at 48 h calculated by the Excel® trendline function (Aboul-Soud et al., 2020) were 269 and 258 µg EECC/ml for MCF-7 and MDA 231 cells, respectively. None of the extracts under investigation had measurable effects (12%; Supplementary Figure S1) on healthy non-transformed breast fibroblast (MCF-12) control cells.

### EECC induces apoptosis in breast cancer cell lines

**FIGURE 2:**
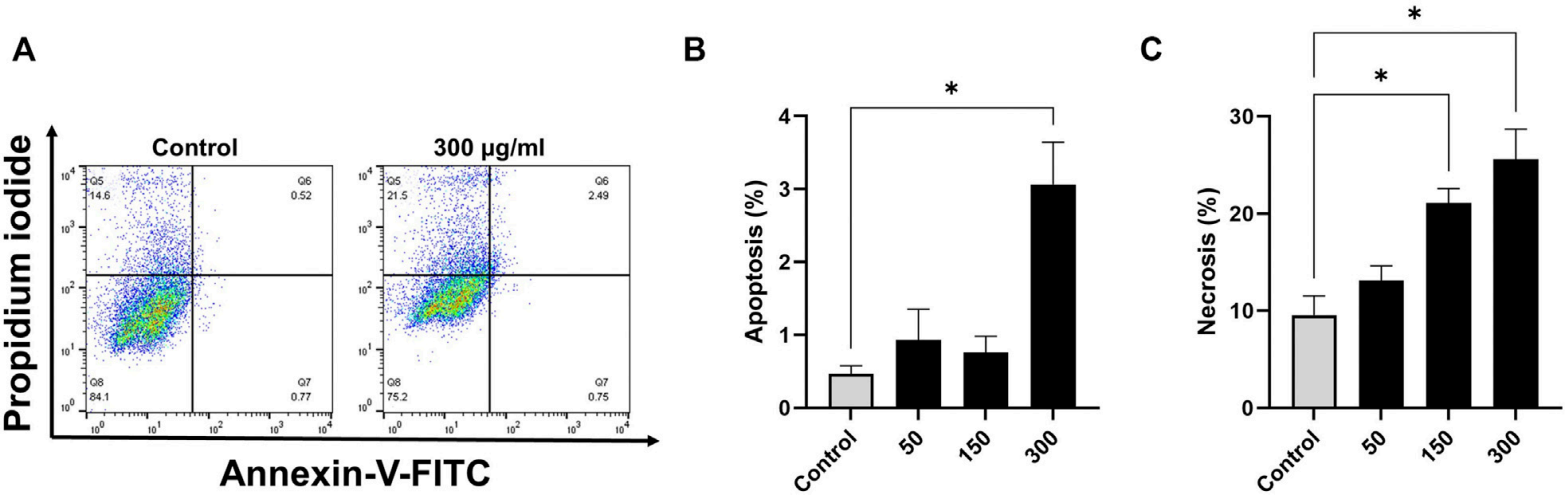
Flow cytometry analysis of MCF-7 cells following the treatment with various concentrations of ethanolic extracts of *C. comosum*. MCF-7 cells were treated with EECC 50, 150 or 300 μg/ml for 48 h. Apoptosis and necrosis were detected by use of FACS Annexin V and PI staining. *p < 0.05 vs. control. Data represent mean ± SD of triplicates.

**FIGURE 3:**
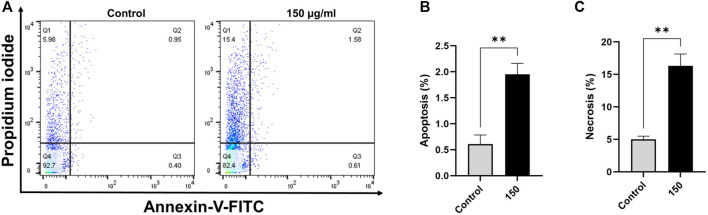
Ethanol extract of *Colligonum comosum* (EECC) induces apoptosis and necrosis in MDA 231 cells. MDA 231 cells were treated with indicated concentrations of EECC for 48 h. Cells were then labeled with Annexin V and PI and analyzed by flow cytometry as described in the Methods section. **p < 0.01 vs. control. Data represent mean ± SD of triplicates.

Based on annexin V-FITC staining for detection of apoptosis, compared with control cells, breast cancer cells exposed to EECC for 48 h exhibited significant increase in apoptosis (Figures 2, 3). A significant positive correlation was evident between dead cells stained with FITC-Annexin/PI for each EECC concentration and its corresponding IC_50_.

MCF-7 breast cancer cells treated with 50, 150 or 300 µg EECC/mL exhibited significant apoptosis as determined by FACS dual Annexin-V/PI staining, with the greatest effect observed for 300 µg EECC/mL. Exposure to 300 µg EECC/ml resulted in significant apoptosis (0.46 ± 0.11% to 3.06 ± 0.57%) (Figures 2A,B). Alternatively, necrosis, was observed at 150 µg EECC/ml (21.09 ± 1.49%) as well as 300 µg EECC/ml (25.60 ± 3.09%) (Figures 2A,C). Similarly, in MDA 231 breast cancer cells, exposure to 150 µg EECC/ml caused significant apoptosis (0.60 ± 0.17% to 1.95 ± 0.21% (Figures 3A,B) and necrotic cells (4.99 ± 0.50% to 16.30 ± 1.83% (Figures 3A,C).

### EECC causes cell cycle arrest

**FIGURE 4:**
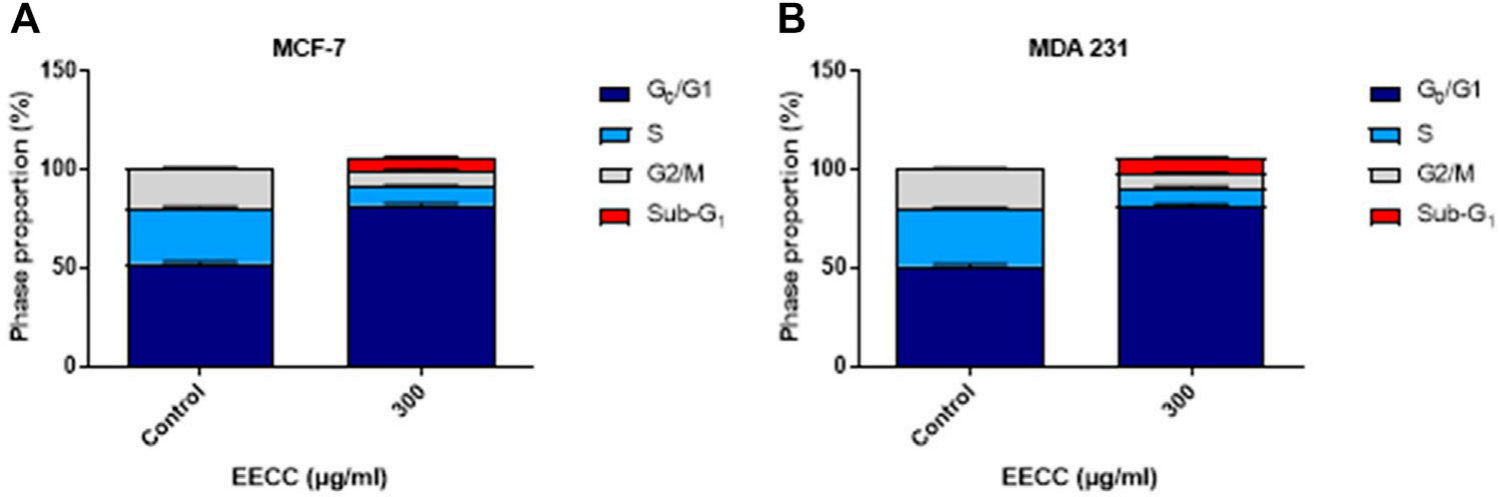
Ethanol extract of *Colligonum comosum* (EECC) causes cell cycle arrest in MCF-7 and MDA 231 cells. Following treatment for 48 h with 300 μg/ml of EECC, cells were analyzed for DNA content as detailed in the Methods section.

In order to examine if 300 μg/ml EECC-induced apoptosis led to disruption in cell cycle progression, MCF-7 and MDA 231 cells were stained with PI and analyzed for DNA content by flow cytometry. A significant increase in the proportion of sub-G_1_, as an indicative marker for the percentage of apoptotic cells, between control and EECC-treated cells was noted in MCF-7 (0.27% ± 0.03 vs. 6.53% ± 0.39) and MDA 231 (0.24 ± 0.02 vs. 7.53 ± 0.39) (Figures 4A,B). Taken together, as Figure 4 indicates, cells were arrested at the G_1_ phase, as indicated by an increased proportion of cells arrested in G_1_ phase upon EECC exposure, along with significant DNA loss evident as increase in sub-G_1_ cells.

### EECC-induced cell death in breast cancer cells is mediated through OS

**FIGURE 5:**
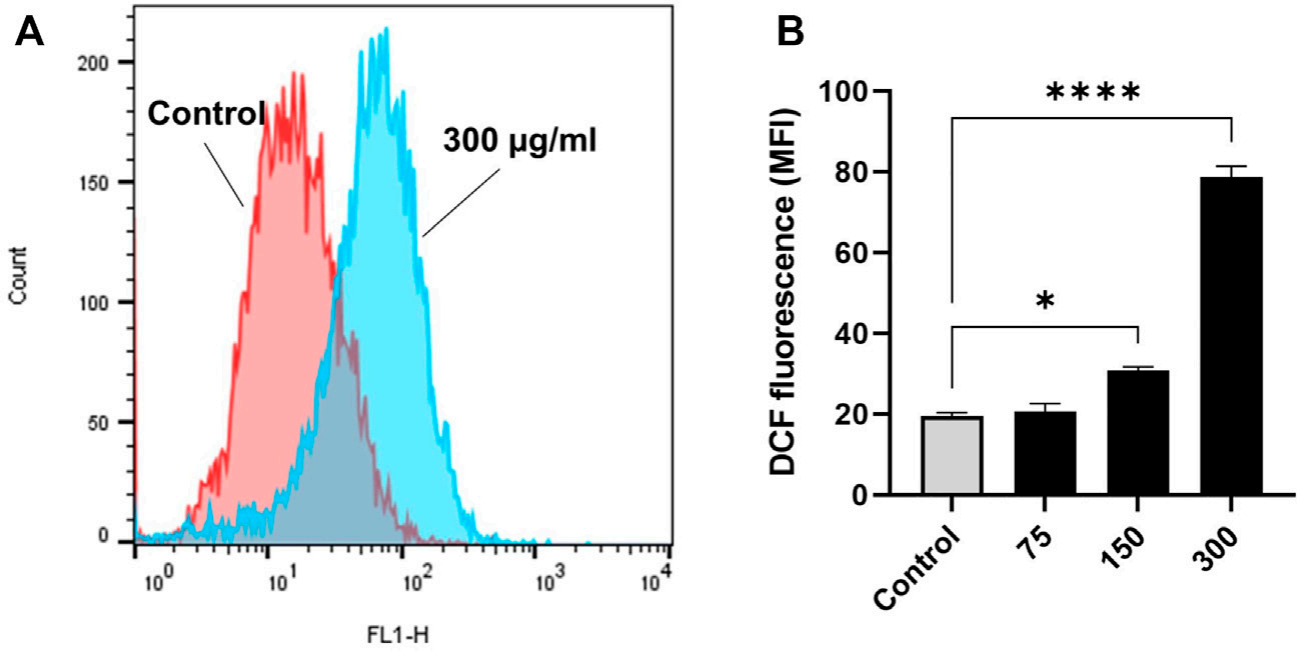
Ethanol extract of *Colligonum comosum* (EECC) causes oxidative stress in MCF-7 cells. MCF-7 cells were treated for 48 h with indicated concentrations of EECC. Cells were analyzed for DCF fluorescence (ROS generation) after having been stained with DCFDA. *p < 0.05, ***p < 0.0001 vs. control. Data represent mean ± SD of triplicates.

Exposure to EECC caused OS as demonstrated by 10 µM H_2_DCFDA of MCF-7 breast cancer cells. Viability of MCF-7 cells was significantly less after a 30 min exposure to 10 µM H_2_DCFDA, relative to untreated control (Figure 5). Since over-production of ROS predisposes cells to premature death, control and experimental cells were stained with H_2_DCFDA to evaluate oxidative damage. Significantly greater fluorescence was observed in cells exposed to 150 or 300 µg EECC/ml, which ranged from 19.5 ± 0.90 a.u. to 30.75 ± 0.95 a.u. and to 78.70 ± 2.7 a.u., respectively (Figures 5A,B).

### EECC stimulates caspase-3 enzyme activity

**FIGURE 6:**
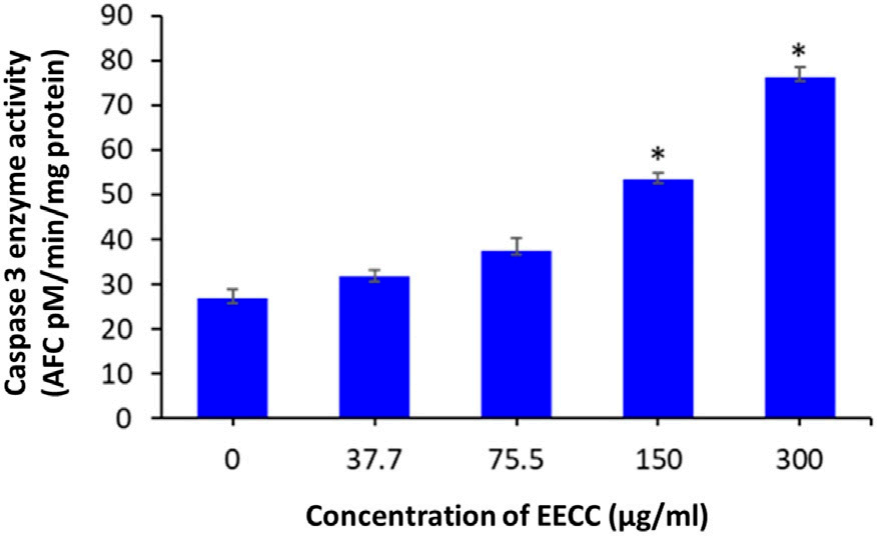
Caspase-3 enzyme activity in MCF- cells following exposure to different concentrations of ethanol extracts of *Calligonum comosum* for 48 h *p < 0.05 vs. control. Data represent mean ± SD of eight technical well-replicates.

Exposure of MCF-7 breast cancer cells to 150 or 300 µg EECC/ml significantly induced caspase-3 enzyme activity relative to control group, but caspase-3 enzymatic activity was low at 37.7 and 75.7 μg/ml compared to control (Figure 6).

### Identification of compounds present in EECC by GC-MS

**FIGURE 7:**
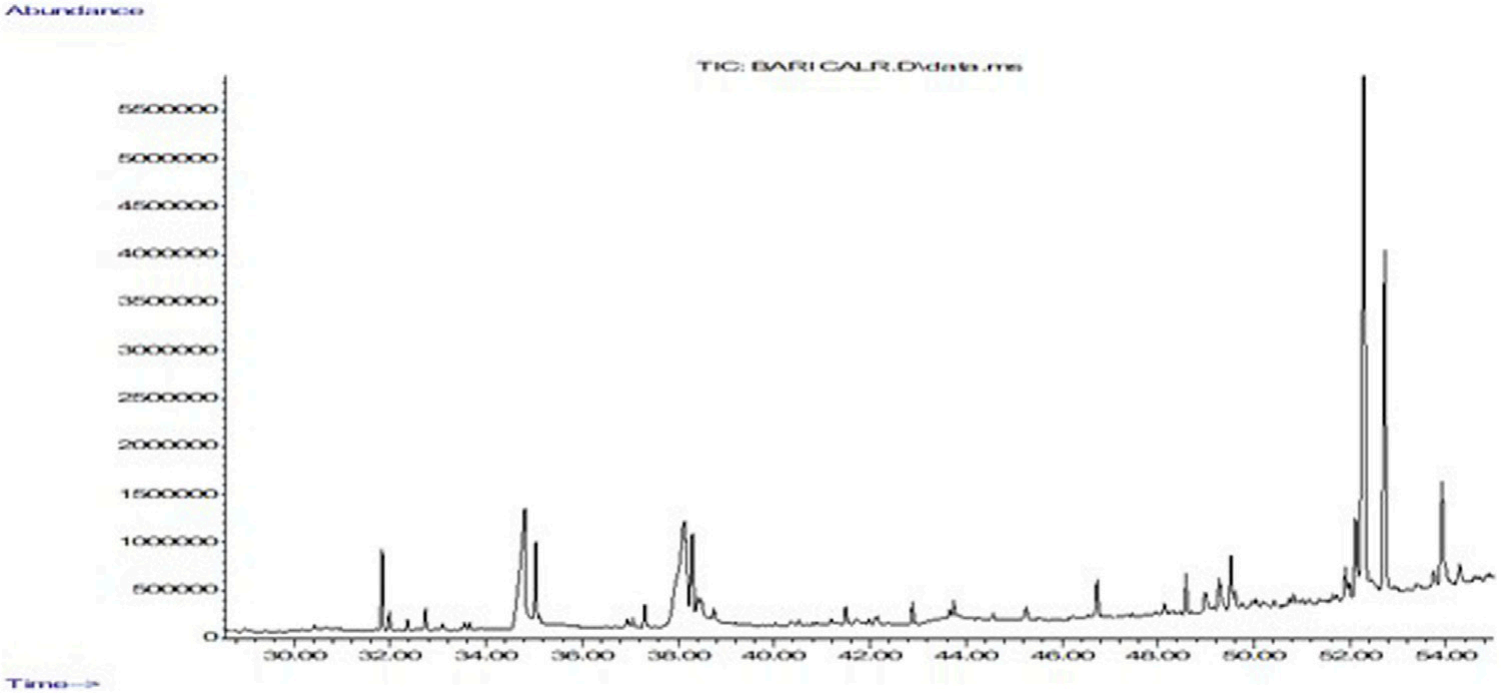
Gas chromatography mass spectrometry analysis of ethanol extract of *Colligonum comosum.* Image depicts a representative chromatograph of constituents identified by GC/MS in in EECC. Peaks represent absolute abundances, whereas numbers on the *x*-axis represent retention times in min.

To identify some of the most prevalent phytochemical present and determine those most likely to cause anti-proliferative activity of EECC against the two breast cancerous cells, EECC was subjected to GC-MS analysis (Table 1), which demonstrated that one of the predominate compounds was hexacosanol which accounted for 33% of the mass of compounds in EECC, followed by tocopherol, which accounted for 14%. Other potentially biologically active molecules like squalene, which is a triterpine was present at 1.47%. Other molecules present included chloroheptacosane (0.45%), campesterol (5.7%), bicyclooheptane (2.74%), hexadecanoic acid (11.2%), ethylester of hexadecanoic acid (2.5%), octadecadienoic acid (13.7%) and methyl ester of octadecadienoic acid (3.1%) (Figure 7).

**TABLE 1:** GC-MS data of ethanol extract of *Colligonum comosum l*eaves.

Compound name	Mol mass (amu)	RT (min)	Area	% Area
Propanoic acid, 2-oxo-, methyl ester	102.032	5.441	490244	0.60
2(4H)-Benzofuranone, 5,6,7,7a-tetrahydro-4,4,7a-trimethyl-, (R)-	180.115	24.597	138677	0.17
Bicyclo[3.1.1]heptane, 2,6,6-trimethyl-, [1R-(1.alpha.,2.beta.,5.alpha.)]-	138.141	31.837	**2215662**	**2.74**
2-Pentadecanone, 6,10,14-trimethyl-	268.277	31.983	515320	0.6
3,7,11,15-Tetramethyl-2-hexadecen-1-ol	296.308	32.365	352794	0.4
1-Hexadecyne	222.235	32.74	620968	0.7
4-(2,2-Dimethyl-6-methylenecyclohexyl)butanal	194.167	33.548	197306	0.2
Hexadecanoic acid, methyl ester	270.256	33.662	205507	0.2
n-Hexadecanoic acid	256.24	34.744	**9070053**	**11.2**
Hexadecanoic acid, ethyl ester	284.272	35.03	**2068153**	**2.5**
Methyl 11,14-octadecadienoate	294.256	36.932	195055	0.2
cis,cis,cis-7,10,13-Hexadecatrienal	234.198	37.06	211698	0.2
Phytol	296.308	37.314	815264	1.0
9,12-Octadecadienoic acid (Z,Z)-	280.24	38.065	**11069891**	**13.7**
9,12,15-Octadecatrienoic acid, ethyl ester, (Z,Z,Z)-	306.256	38.294	**2522968**	**3.1**
Octadecanoic acid	284.272	38.408	280858	0.3
Methyl 17-methyl-octadecanoate	312.303	38.752	285390	0.3
n-Tetracosanol-1	354.386	40.374	158654	0.19
Eicosane	282.329	40.521	139890	0.17
4,8,12,16-Tetramethylheptadecan-4-olide	324.303	41.494	475893	0.59
Nonanoic acid, 2,4,6-trimethyl-, methyl ester, (2S,4R,6R)-(+)-	214.193	42.118	195836	0.24
1-Docosene	308.344	43.657	162588	0.20
Heptacosane, 1-chloro-	414.399	43.746	364875	0.45
3-(3,6-Dimethyl-3,6-dihydro-2H-pyran-2-yl)-3-hydroxy-3H-isobenzofuran-1-one	260.105	44.567	137774	0.17
Octadecane	254.297	45.254	425186	0.52
Ascorbyl Palmitate	414.262	48.136	232827	0.29
Squalene	410.391	48.581	**1189816**	**1.47**
Docosane	310.36	49.516	**1498739**	**1.85**
Stigmastan-3,5,22-trien	394.36	51.909	698736	0.86
1-Hexacosanol	382.417	52.284	**26610812**	**33.01**
dl-.alpha.-Tocopherol	430.381	52.723	**11895720**	**14.75**
p-Toluic acid, heptadecyl ester	374.318	53.76	303547	0.37
Campesterol	400.371	53.932	**4571694**	**5.7**
Stigmasterol	412.371	54.301	284535	0.35

### Molecular docking *of extracts of leaves of Calligonum comosum*

**FIGURE 8:**
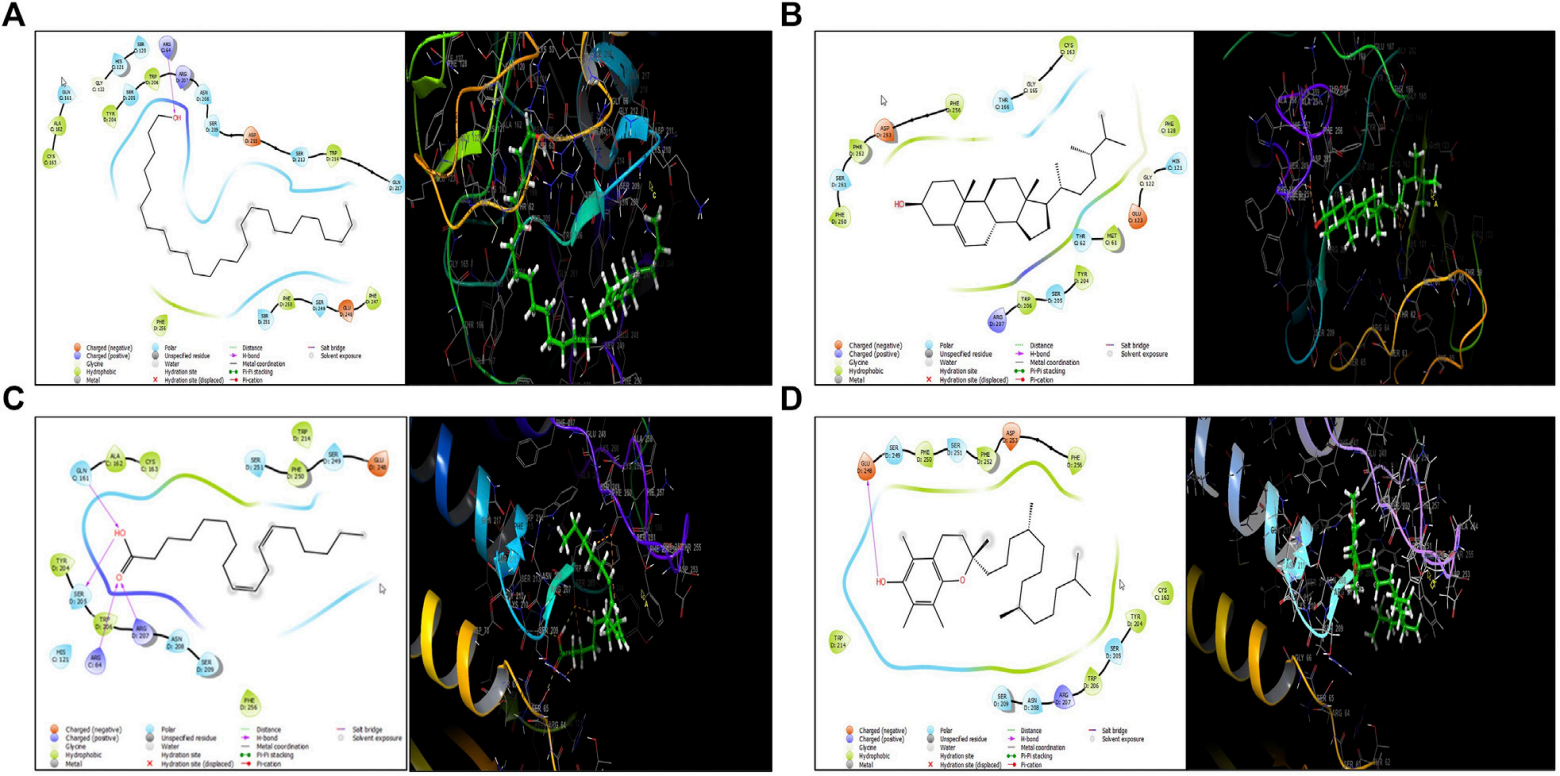
Molecular docking of four major polyphenolics to caspase-3. **(A)** The 2D (left) and 3D (right) diagrams of the 1-Hexacosanol interactions with the active site of caspase-3 (PDB: 3GJQ). **(B)** The 2D (left) and 3D (right) diagrams of the 9,12-Octadecadienoic acid (Z,Z)- interactions with the active site of caspase-3 (PDB: 3GJQ). **(C)** The 2D (left) and 3D (right) diagrams of the Campesterol interactions with the active site of caspase-3 (PDB: 3GJQ). **(D)** The 2D (left) and 3D (right) diagrams of the DL-alpha-Tocopherol interactions with the active site of caspase-3 (PDB: 3GJQ).

To investigate their binding interaction of with different amino acids in the active site of caspase 3, four of the major phytoconstituents found in EECC were docked. DL-alpha-Tocopherol, Campesterol, 1-Hexacosanol and 9,12-Octadecadienoic acid (Z,Z) had significant potential as ligands in the binding domain of the caspase-3 receptor (PDB:3GJQ) with glide scores of −4.142, −3.536, −2.637 and −0.957 kcal/mol, respectively (Table 2). The 2D and 3D diagrams depicting the molecular interactions of the selected four compounds having the highest glide scores (i.e., greatest binding affinity in terms of energy values in kcal/mol) with the active site of caspase-3 are shown in Figure 8.

**TABLE 2:** Docking results with ligands in the caspase-3 (PDB:3GJQ) receptor.

Compound name	Compound ID	Formula	Glide score (kcal/mol)	Glide energy (kcal/mol)	Glide emodel (kcal/mol)
1-Hexacosanol	68171	C_26_H_54_O	−2.637	−39.887	−44.301
9,12-Octadecadienoic acid (Z,Z)-	5280450	C_18_H_32_O_2_	−0.957	−38.729	−37.825
Campesterol	173183	C_28_H_48_O	−3.536	−29.978	−34.135
DL-alpha-Tocopherol	2116	C_29_H_50_O_2_	−4.142	−35.413	−43.364

## Discussion

Plants have indeed been studied all around the world to determine whether they might contain novel anticancer drugs with minimal adverse effects on non-target cells. Recent literature has reported effects of various herbal plants for treatment of a variety of illnesses, including cancer. Numerous formulations of plant products are currently available to treat cancer patients. But, mostly chemotherapeutic drugs were also cytotoxic to non-cancerous cells and could result in resistance of targeted cells to the drug (Singh et al., 2014). Thus, investigation of conventionally used herbs or plant products for the management of various cancers could be regarded as a useful source for novel therapeutic agents (Alsughayyir et al., 2022). Several investigations, conducted in the past few decades to find compounds that are effective at preventing and treating breast cancer. These have culminated into the discovery of herbal and plant products and recipes that can influence the onset, development, and advancement of breast cancer (El-Shemy et al., 2010; Aboul-Soud et al., 2021). One example is tamoxifen, which is an estrogen receptor antagonist, present in the Pacific yew (*Taxus brevifolia*), that is used to treat, among other strategies, breast cancer (Mahmoud et al., 2016).

It has been reported that *Calligonum comosum* exhibits antihyperglycemic activities, anti-inflammatory, anti-ulcer and anticancer activities in both rat and shrimp (El-Hawary and Kholief, 1990; Liu et al., 2001). In another study, *Calligonum comosum* exhibited both cytotoxicity and genotoxicity to human hepatocellular carcinoma (HepG2) cells (Shalabi et al., 2015; Alzahrani, 2021) and human triple-negative MDA-MB-231 breast cancer cells through the apoptotic pathway (Alehaideb et al., 2020). In HepG2 cells,extracts of *Calligonum comosum* have been reported to induce cytotoxicity through the apoptotic pathway (Shalabi et al., 2015; Alzahrani, 2021). Thus, in this study it was clarified whether EECC inhibited growth of the breast cancer cells *via* apoptosis. When MCF-7 and MDA 231 cells were exposed to EECC for 48 h, followed by incubation with Annexin V and PI as described in Methods it was determined that EECC had initiated apoptosis and necrosis in a dose-dependent manner in MCF7 cells. No cell death was observed at a concentration 50 µg EECC/mL. However, at 150 and 300 µg EECC/ml increases in both apoptosis and necrosis were observed with the greater effect in MCF7 cells exposed to 300 μg/ml. Alternatively, exposure to 150 µg EECC/mL resulted in significant apoptosis and necrosis in MDA cells, which indicated that effects of EECC are not only dependent on dose, but also cell line. Exposure of both MCF-7 and MDA-MB-231 breast cancer cells to EECC for 48 h stimulated apoptosis. Apoptotic cells may progress into necrosis (termed secondary necrosis) especially *in vitro* with the lack of phagocytes. In the case of EECC, it is apparent that the relatively higher increase in the percentage of necrotic cells compared to that of apoptotic cells suggests secondary necrosis (i.e., cells that have gone through apoptosis and then became necrotic) (Silva, 2016; Rogers et al., 2017; Sachet et al., 2017). A time-course experiment to monitor apoptotic/necrotic cells over time might confirm or negate this assumption. Importantly, the rest of the data presented in Figures 2–6 strongly suggest the presence of apoptotic transformation including ROS accumulation, cell cycle arrest, and caspase activation. Taken together, these results indicate that EECC inhibits growth of breast cancer cells, in part, by initiating apoptosis. These results are consistent with results of MTT assays (Figure 1) and further validate previous reports that extracts of *Calligonum comosum* induce apoptosis in various *in vitro* human cancers (Shalabi et al., 2015; Alehaideb et al., 2020; Alzahrani, 2021). Although slight but significant apoptosis was observed, necrotic cell death was far more profound. This, however, might not be interpreted as primary necrosis given the *in vitro* nature of this study and the lack of phagocytes. Failure to dispose of apoptotic cells, results in progression of apoptotic cells into secondary necrosis with ruptured plasma membrane. Thus, treated cells might have initially undergone apoptosis which was then followed by necrosis, or, alternatively, a proportion of the cells may have become directly necrotic upon exposure. In all cases, both apoptosis and necrosis were observed which is consistent with other findings in the current report.

Data of the studies, results of which are presented here, also show that EECC caused cell cycle arrest in both cellular models (Figure 4). Inhibition of cell cycle progression is a pivotal mechanism of anticancer therapeutics (Bai et al., 2017) to limit the growth and spread of tumor cells. Cell division is arrested when the cell undergoes overwhelming physical or chemical stress that outweighs its ability to repair damaged DNA (Toettcher et al., 2009). Thus, it seems plausible that EECC also caused significant fragmentation of DNA, typical of apoptotic transformation (Alfhili et al., 2021c). Further examination of damage to DNA and cell cycle regulatory proteins, most notably, p21, p27, cyclins, and cylcin-dependent kinases (CDKs) is warranted. Importantly, if and how EECC modulates transcription and translation of DNA repair genes, possibly through microRNA, long-noncoding RNA, or epigenetic modification, remains to be investigated.

ROS are known to cause manifold and frequently inconsistent effects in cells (Galadari et al., 2017). If ROS molecules are not scavenged from tissues or reduced, they increase the risk of breast development by damaging nucleic acid, lipids and proteins. When cellular production of ROS was evaluated in MCF-7 cells exposed for 30 min to several concentrations of EECC generation of ROS was 80% greater than that observed in control cells. Exposure to either 150 or 300 μg EECC/ml, resulted in significantly greater amounts of cellular ROS, relative to the cellular ROS produced in the control cells. ROS can cause oxidative reactions in cells. This causes cell cycle arrest, inflammation, and cell death, which may be followed by apoptosis, necrosis, autophagy, or consequences controlled by intersecting networks (Scherz-Shouval and Elazar, 2011; Lee et al., 2012). Recently it has been shown that ROS contribute premature red cell death possibly by modulating intracellular Ca^2+^ homeostasis (Alfhili et al., 2021a). When cytosolic Ca^2+^ increases, the activity of enzymes responsible for the movement of phospholipids in the cell membrane is impaired., resulting in phosphatidylserine externalization. ROS may lead to excessive activity of Ca^2+^ channels thereby raising Ca^2+^ stores upstream of subsequent loss of membrane asymmetry. A modest amount of ROS plays a key role in apoptosis, as well as survival and differentiation of cells (Redza-Dutordoir and Averill-Bates, 2016). Taken together, these results demonstrate that exposure of MCF-7 cells to EECC leads to increased cell death, which most properly is the effect from increased ROS activity, hence EECC uses ROS to induce cell death in these cancer cells by increasing ROS concentrations; known triggers of cell death (Alfhili et al., 2021b).

Expression of caspase-3, an executioner enzyme in apoptosis, was significantly greater after exposure to 150 or 300 µg EECC/ml compared to unexposed, control cells. Alternatively, exposure to 37.7 or 75.7 µg EECC/ml resulted in lesser expression of caspase-3 compared to control. In the study, results of which are reported here, EECC was quantified by use of gas chromatography coupled with mass spectroscopy (GC-MS) to identify and quantify the effective molecules present in EECC including alcohols, alkaloids, nitro compounds, longer-chain hydrocarbons, organic acids, steroids, esters and amino acids (Table 1 and Figure 6). EECC contained smaller and larger esters, terpenes, conjugated acid, fatty alcohol and various acids. Among the compounds identified by GC-MS, molecular docking was done only for major compounds such as 1-Hexacosanol, 9,12-Octadecadienoic acid (Z,Z)-, Campesterol, DL-alpha-Tocopherol, n-Hexadecanoic acid. According to the results obtained, DL-alpha-tocopherol was the most active compound as it had a better glide score (−4.142 kcal/mol). Additionally, DL-alpha-tocopherol formed a hydrophobic contact with Glu 248 (Figure 8 and Table 2).

## Conclusion

Based on the results of this study and information available in the literature, it can be concluded that EECC inhibits proliferation of breast cancer cells by causing OS-mediated apoptosis. Viability of breast cancer cells were found to decrease in a concentration-dependent manner due to EECC when exposed for 48 h. Results of FACS demonstrated that EECC caused apoptosis and necrosis in a dose-dependent manner on both breast cancer cells under investigation. OS is thus considered as a major mechanism of toxicity behind that caused by EECC exposure. EECC also stimulates the apoptotic pathway by up-regulation of casapase-3 in treated breast cancer cells. Results of this study indicated that EECC has potential for use as an alternative therapeutic agent to treat cancer (Krishan, 1975; Murray, 1992; Silva, 2010; Khan et al., 2018).

## Data Availability

The original contributions presented in the study are included in the article/Supplementary Material, further inquiries can be directed to the corresponding author.

## References

[B1] Aboul-Soud M. A. M. Ennaji H. Kumar A. Alfhili M. A. Bari A. Ahamed M. (2022). Antioxidant, anti-proliferative activity and chemical fingerprinting of *Centaurea calcitrapa* against breast cancer cells and molecular docking of caspase-3. Antioxidants (Basel) 11 (8), 1514. 10.3390/antiox11081514 36009233 PMC9405406

[B2] Aboul-Soud M. A. M. Ashour A. E. Challis J. K. Ahmed A. F. Kumar A. Nassrallah A. (2020). Biochemical and molecular investigation of in vitro antioxidant and anticancer activity spectrum of crude extracts of willow leaves salix safsaf. Plants 9, 1295. 10.3390/plants9101295 PMC7599573 33008079

[B3] Adams R. P. (2007). Identification of essential oil components by gas chromatography/ mass spectrometry. 4th Edition. Carol Stream, IL: Allured Publ.

[B4] Akhtar M. J. Ahamed M. Alhadlaq H. (2020). Gadolinium oxide nanoparticles induce toxicity in human endothelial huvecs via lipid peroxidation, mitochondrial dysfunction and autophagy modulation. Nanomaterials 10, 1675. 10.3390/nano10091675 PMC7559735 32859033

[B5] Alehaideb Z. AlGhamdi S. Yahya W. B. Al-Eidi H. Alharbi M. Alaujan M. (2020). Anti-proliferative and pro-apoptotic effects of *Calligonum comosum* (L’her.) methanolic extract in human triple-negative MDA-MB-231 breast cancer cells. J. Evid. Based. Integr. Med. 25, 2515690X20978391. 10.1177/2515690X20978391 PMC7734547 33302699

[B6] Alfhili M. A. Alsalmi E. Aljedai A. Alsughayyir J. Abudawood M. Basudan A. M. (2021a). Calcium-oxidative stress signaling axis and casein kinase 1α mediate eryptosis and hemolysis elicited by novel p53 agonist inauhzin. J. Chemother. 34, 247–257. Online ahead of print. 10.1080/1120009X.2021.1963616 34410893

[B7] Alfhili M. A. Basudan A. M. Aljaser F. S. Dera A. Alsughayyir J. (2021b). Bioymifi, a novel mimetic of TNF-related apoptosis-induced ligand (TRAIL), stimulates eryptosis. Med. Oncol. 38 (12), 138. 10.1007/s12032-021-01589-5 34633592

[B8] Alfhili M. A. Hussein H. A. M. Park Y. Lee M. H. Akula S. M. (2021c). Triclosan induces apoptosis in Burkitt lymphoma-derived BJAB cells through caspase and JNK/MAPK pathways. Apoptosis. 26 (1-2), 96–110. 10.1007/s10495-020-01650-0 33387145

[B9] Alsughayyir J. Alshaiddi W. Alsubki R. Alshammary A. Basudan A. M. Alfhili M. A. (2022). Geraniin inhibits whole blood IFN-γ and IL-6 and promotes IL-1β and IL-8, and stimulates calcium-dependent and sucrose-sensitive erythrocyte death. Toxicol. Appl. Pharmacol. 436, 115881. 10.1016/j.taap.2022.115881 35026210

[B10] Alzahrani A. J. (2021). Potent antioxidant and anticancer activities of the methanolic extract of *Calligonum comosum* (L’Her) fruit hairs against human hepatocarcinoma cells. Saudi J. Biol. Sci. 28 (9), 5283–5289. 10.1016/j.sjbs.2021.05.053 34466106 PMC8380993

[B11] Ashour A. E. Ahmed A. F. Kumar A. Zoheir K. M. Aboul-Soud M. A. Ahmad S. F. (2016). Thymoquinone inhibits growth of human medulloblastoma cells by inducing oxidative stress and caspase-dependent apoptosis while suppressing NF-B signaling and IL-8 expression. Mol. Cell. Biochem. 416, 141–155. 10.1007/s11010-016-2703-4 27084536

[B12] Badria F. A. Ameen M. Akl M. R. (2007). Evaluation of cytotoxic compounds from calligonum comosum L. growing in Egypt. Z. Naturforsch. C J. Biosci. 62, 656–660. 10.1515/znc-2007-9-1005 18069236

[B13] Bai J. Li Y. Zhang G. (2017). Cell cycle regulation and anticancer drug discovery. Cancer Biol. Med. 14 (4), 348–362. 10.20892/j.issn.2095-3941.2017.0033 29372101 PMC5785171

[B14] Balamurugan R. Stalin A. Ignacimuthu S. (2012). Molecular docking of γ-sitosterol with some targets related to diabetes. Eur. J. Med. Chem. 47, 38–43. 10.1016/j.ejmech.2011.10.007 22078765

[B15] Boulos L. (2000). Flora of Egypt. Cairo, Egypt: Al Hadara Publishing Inc.

[B16] Chebbac K. Ghneim H. K. El Moussaoui A. Bourhia M. El Barnossi A. Benziane Ouaritini Z. (2022). Antioxidant and antimicrobial activities of chemically-characterized essential oil from *Artemisia aragonensis* lam. Molecules 27 (3), 1136. 10.3390/molecules27031136 35164402 PMC8840534

[B17] Chen Z. Zhang B. Gao F. Shi R. (2018). Modulation of G_2_/M cell cycle arrest and apoptosis by luteolin in human colon cancer cells and xenografts. Oncol. Lett. 15 (2), 1559–1565. 10.3892/ol.2017.7475 29434850 PMC5776168

[B18] Cheng Y. Y. Hsieh C. H. Tsai T. H. (2018). Concurrent administration of anticancer chemotherapy drug and herbal medicine on the perspective of pharmacokinetics. J. Food Drug Anal. 26 (2S), S88–S95. 10.1016/j.jfda.2018.01.003 29703390 PMC9326883

[B19] El-Hawary Z. Kholief T. (1990). Biochemical studies on some hypoglycemic agents (II) effect of *Calligonum comosum* extract. Arch. Pharm. Res. 13, 113–116. 10.1007/bf02857846

[B20] El-Shemy H. A. Aboul-Soud M. A. Nassr-Allah A. A. Aboul-Enein K. M. Kabash A. Yagi A. (2010). Antitumor properties and modulation of antioxidant enzymes’ activity by *Aloe vera* leaf active principles isolated via supercritical carbon dioxide extraction. Curr. Med. Chem. 17 (2), 129–138. 10.2174/092986710790112620 19941474

[B21] El-Sheref E. M. Aly A. A. Alshammari M. B. Brown A. B. Abdel-Hafez S. M. N. Abdelzaher W. Y. (2020). Design, synthesis, molecular docking, antiapoptotic and caspase-3 inhibition of new 1, 2, 3-triazole/bis-2 (1H)-quinolinone hybrids. Molecules 25 (21), 5057. 10.3390/molecules25215057 PMC7672604 33143331

[B22] Friesner R. A. Murphy R. B. Repasky M. P. Frye L. L. Greenwood J. R. Halgren T. A. (2006). Extra precision glide: Docking and scoring incorporating a model of hydrophobic enclosure for protein− ligand complexes. J. Med. Chem. 49 (21), 6177–6196. 10.1021/jm051256o 17034125

[B23] Galadari S. Rahman A. Pallichankandy S. Thayyullathil F. (2017). Reactive oxygen species and cancer paradox: To promote or to suppress? Free Radic. Biol. Med. 104, 144–164. 10.1016/j.freeradbiomed.2017.01.004 28088622

[B24] Ghazanfar S. A. (1994). Handbook of Arabian medicinal plants. Boca Raton: CRC Press, 173.

[B25] Ghoncheh M. Pournamdar Z. Salehiniya H. (2016). Incidence and mortality and epidemiology of breast cancer in the world. Asian pac. J. Cancer Prev. 17 (S3), 43–46. 10.7314/apjcp.2016.17.s3.43 27165206

[B26] Iqbal J. Abbasim B. A. Mahmood T. Kanwal S. Ali B. Shah S. A. (2017). Plant-derived anticancer agents: A green anticancer approach. Asian pac. J. Trop. Biomed. 7 (12), 1129–1150. 10.1016/j.apjtb.2017.10.016

[B27] Khan S. Zafar A. Naseem I. (2018). Copper-redox cycling by coumarin-di(2-picolyl)amine hybrid molecule leads to ROS-mediated DNA damage and apoptosis: A mechanism for cancer chemoprevention. Chem. Biol. Interact. 290, 64–76. 10.1016/j.cbi.2018.05.010 29803612

[B28] Krishan A. (1975). Rapid flowcytofluorometric analysis of mammalian cell cycle by propidium iodide staining. J. Cell Biol. 66, 188–193. 10.1083/jcb.66.1.188 49354 PMC2109516

[B29] Lee J. Giordano S. Zhang J. (2012). Autophagy, mitochondria and oxidative stress: Cross-talk and redox signalling. Biochem. J. 441, 523–540. 10.1042/BJ20111451 22187934 PMC3258656

[B30] Liu X. M. Zakaria M. N. Islam M. W. Radhakrishnan R. Ismail A. Chen H. B. (2001). Anti-inflammatory and anti-ulcer activity of Calligonum comosum in rats. Fitoterapia 72 (5), 487–491. 10.1016/s0367-326x(01)00271-4 11429240

[B31] Mahmoud A. M. Aboul-Soud M. A. Han J. Al-Sheikh Y. A. Al-Abd A. M. El-Shemy H. A. (2016). Transcriptional profiling of breast cancer cells in response to mevinolin: Evidence of cell cycle arrest, DNA degradation and apoptosis. Int. J. Oncol. 48 (5), 1886–1894. 10.3892/ijo.2016.3418 26983896 PMC4809649

[B32] Mitra S. Dash R. (2018). Natural products for the management and prevention of breast cancer. Evid. Based. Complement. Altern. Med. 2018, ID8324696. 10.1155/2018/8324696 PMC5846366 29681985

[B33] Murray A. W. (1992). Creative blocks: Cell-cycle checkpoints and feedback controls. Nature 359, 599–604. 10.1038/359599a0 1406993

[B34] Redza-Dutordoir M. Averill-Bates D. A. (2016). Activation of apoptosis signalling pathways by reactive oxygen species. Biochim. Biophys. Acta 1863 (12), 2977–2992. 10.1016/j.bbamcr.2016.09.012 27646922

[B35] Riadh H. Imen F. Abdelmajid Z. Sinda F. (2011). Detection and extraction of Calligonum comosum, a medicinal plant from arid region of Tunisia. Afr. J. Traditional Complementary Altern. Med. 8 (3), 322–327. PMC3252229 22468012

[B36] Rogers C. Fernandes-Alnemri T. Mayes L. Alnemri D. Cingolani G. Alnemri E. S. (2017). Cleavage of DFNA5 by caspase-3 during apoptosis mediates progression to secondary necrotic/pyroptotic cell death. Nat. Commun. 8, 14128. 10.1038/ncomms14128 28045099 PMC5216131

[B37] Roy A. Anand A. Garg S. Khan M. S. Bhasin S. Asghar M. N. (2022). Structure-based in silico investigation of agonists for proteins involved in breast cancer. Evid. Based. Complement. Altern. Med. 2022, 7278731. 10.1155/2022/7278731 PMC8758269 35035508

[B38] Sachet M. Liang Y. Y. Oehler R. (2017). The immune response to secondary necrotic cells. Apoptosis 22 (10), 1189–1204. 10.1007/s10495-017-1413-z 28861714 PMC5630647

[B39] Safwat G. M. Hassanin K. M. A. Mohammed E. T. Ahmed E. K. Abdel Rheim M. R. Ameen M. A. (2021). Synthesis, anticancer assessment, and molecular docking of novel chalcone-thienopyrimidine derivatives in HepG2 and MCF-7 cell lines. Oxid. Med. Cell. Longev. 2021, 4759821. 10.1155/2021/4759821 35003514 PMC8728392

[B40] Scherz-Shouval R. Elazar Z. (2011). Regulation of autophagy by ROS: Physiology and pathology. Trends biochem. Sci. 36, 30–38. 10.1016/j.tibs.2010.07.007 20728362

[B41] Schwartsmann G. BrondAni dA RochA A. Berlinck R. G. Jimeno J. (2001). Marine organisms as a source of new anticancer agents. Lancet. Oncol. 2, 221–225. 10.1016/s1470-2045(00)00292-8 11905767

[B42] Shalabi M. Khilo K. Zakaria M. M. Elsebaei M. G. Abdo W. Awadin W. (2015). Anticancer activity of Aloe vera and Calligonum comosum extracts separetely on hepatocellular carcinoma cells. Asian pac. J. Trop. Biomed. 5 (5), 375–381. 10.1016/s2221-1691(15)30372-5

[B43] Silva M. T. (2010). Secondary necrosis: The natural outcome of the complete apoptotic program. FEBS Lett. 584 (22), 4491–4499. 10.1016/j.febslet.2010.10.046 20974143

[B44] Singh S. Singh P. P. Roberts L. R. Sanchez W. (2014). Chemopreventive strategies in hepatocellular carcinoma. Nat. Rev. Gastroenterol. Hepatol. 11 (1), 45–54. 10.1038/nrgastro.2013.143 23938452 PMC4334449

[B45] Toettcher J. E. Loewer A. Ostheimer G. J. Yaffe M. B. Tidor B. Lahav G. (2009). Distinct mechanisms act in concert to mediate cell cycle arrest. Proc. Natl. Acad. Sci. U. S. A. 106 (3), 785–790. 10.1073/pnas.0806196106 19139404 PMC2630102

[B46] Varghese E. Samuel S. M. Abotaleb M. Cheema S. Mamtani R. Busselberg D. (2018). The "yin and yang" of natural compounds in anticancer therapy of triple-negative breast cancers. Cancers (Basel) 10 (10), 346. 10.3390/cancers10100346 PMC6209965 30248941

[B47] Zafar A. Singh S. Naseem I. (2017). Cytotoxic activity of soy phytoestrogen coumestrol against human breast cancer MCF-7 cells: Insights into the molecular mechanism. Food Chem. Toxicol. 99, 149–161. 10.1016/j.fct.2016.11.034 27913286

