# Supplementary figures and images for: Apoptosis-mediated anti-proliferative activity of Calligonum comosum against human breast cancer cells, and molecular docking of its major polyphenolics to Caspase-3
Source: Front Cell Dev Biol. 2022 Oct 10;10:972111. doi: 10.3389/fcell.2022.972111 (PMC9588914; doi:10.3389/fcell.2022.972111)

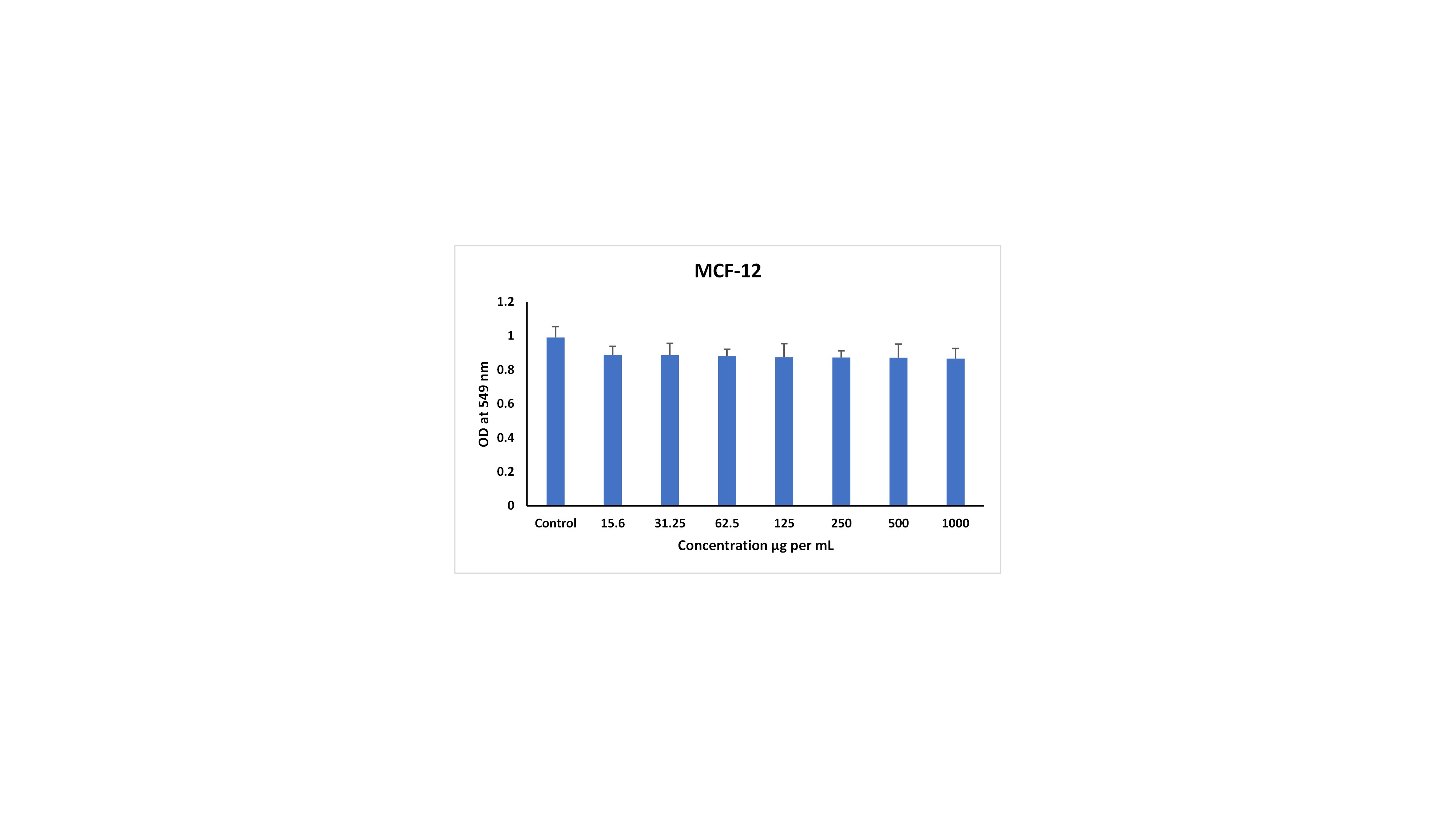

Supplement: Supplementary file 1 [file Image1.JPEG]
